# Supplementary figures and images for: Epidemiology and molecular characterization of Feline panleukopenia virus from suspected domestic cats in selected Bangladesh regions
Source: PLoS One. 2023 Oct 20;18(10):e0282559. doi: 10.1371/journal.pone.0282559 (PMC10588828; doi:10.1371/journal.pone.0282559)

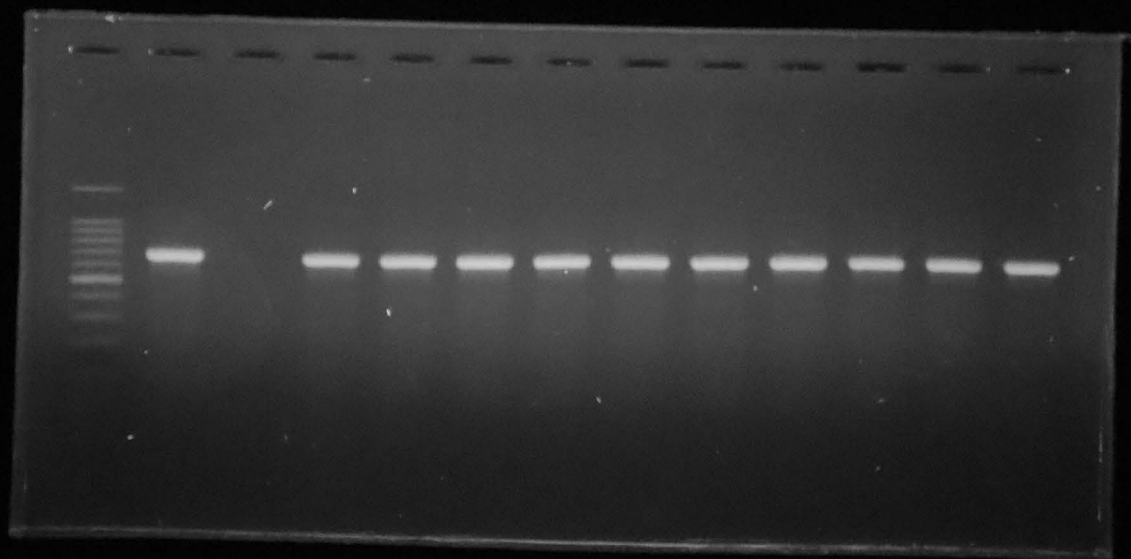

ISO 12800  
Exp. Time: 1/125  
Aperture: 5.6

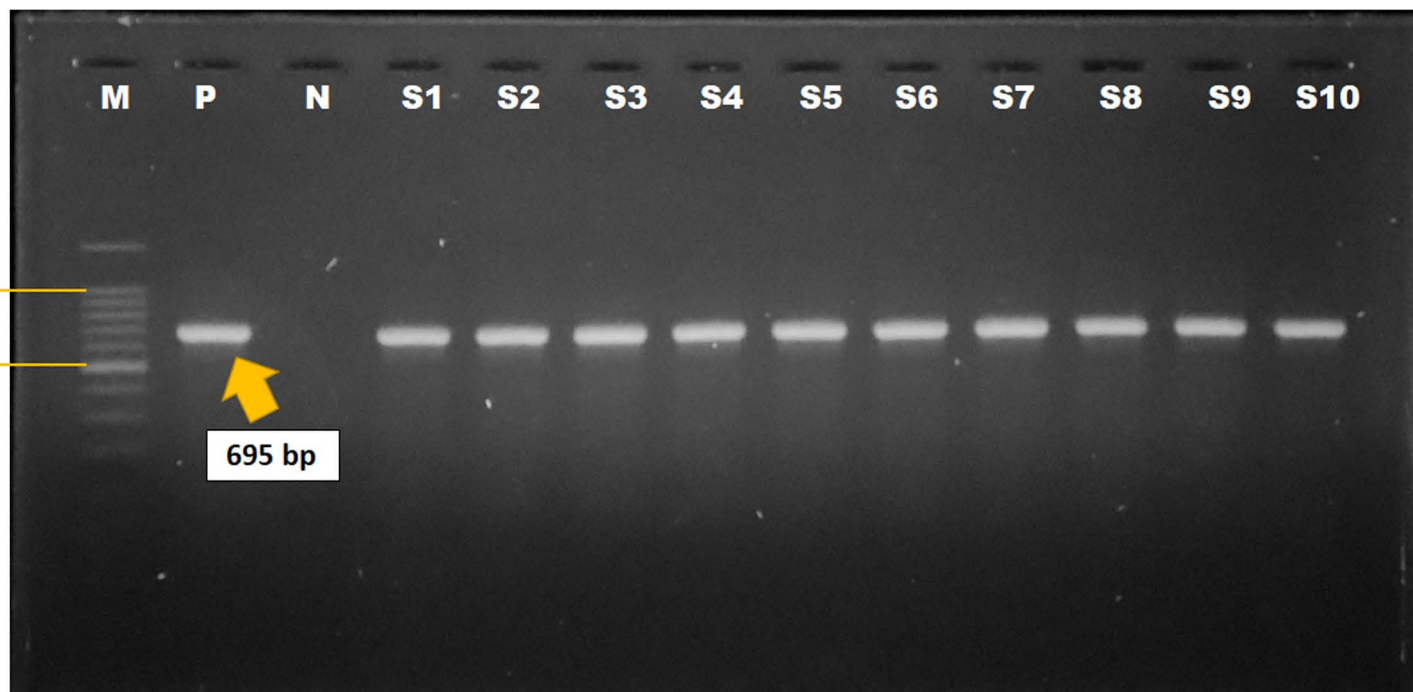

Supplement: S1 Raw images — (PDF) [file pone.0282559.s001.pdf]
